# The incidence, duration, risk factors, and age-based variation of missed opportunities to diagnose pertussis: A population-based cohort study

**DOI:** 10.1017/ice.2023.31

**Published:** 2023-10

**Authors:** Nicholas J. Evans, Alan T. Arakkal, Joseph E. Cavanaugh, Jason G. Newland, Philip M. Polgreen, Aaron C. Miller

**Affiliations:** 1 Department of Internal Medicine, University of Iowa, Iowa City, Iowa; 2 Department of Biostatistics, University of Iowa, Iowa City, Iowa; 3 Department of Pediatrics, Washington University in St. Louis, St. Louis, Missouri

## Abstract

**Objective:**

To estimate the incidence, duration and risk factors for diagnostic delays associated with pertussis.

**Design:**

We used longitudinal retrospective insurance claims from the Marketscan Commercial Claims and Encounters, Medicare Supplemental (2001–2020), and Multi-State Medicaid (2014–2018) databases.

**Setting:**

Inpatient, emergency department, and outpatient visits.

**Patients:**

The study included patients diagnosed with pertussis (*International Classification of Diseases* [ICD] codes) and receipt of macrolide antibiotic treatment.

**Methods:**

We estimated the number of visits with pertussis-related symptoms before diagnosis beyond that expected in the absence of diagnostic delays. Using a bootstrapping approach, we estimated the number of visits representing a delay, the number of missed diagnostic opportunities per patient, and the duration of delays. Results were stratified by age groups. We also used a logistic regression model to evaluate potential factors associated with delay.

**Results:**

We identified 20,828 patients meeting inclusion criteria. On average, patients had almost 2 missed opportunities prior to diagnosis, and delay duration was 12 days. Across age groups, the percentage of patients experiencing a delay ranged from 29.7% to 37.6%. The duration of delays increased considerably with age from an average of 5.6 days for patients aged <2 years to 13.8 days for patients aged ≥18 years. Factors associated with increased risk of delays included emergency department visits, telehealth visits, and recent prescriptions for antibiotics not effective against pertussis.

**Conclusions:**

Diagnostic delays for pertussis are frequent. More work is needed to decrease diagnostic delays, especially among adults. Earlier case identification may play an important role in the response to outbreaks by facilitating treatment, isolation, and improved contact tracing.

Pertussis is a respiratory infection caused by the fastidious, gram-negative coccobacillus *Bordetella pertussis*.^1^ Pertussis is associated with a long-lasting cough. It is spread person to person primarily through respiratory droplets and is extremely contagious among susceptible individuals.^2–5^ Historically, pertussis was a common childhood illness.^6^ Until the 1940s, ∼250,000 cases were reported annually, with a mortality rate approaching 10%.^7^ After the introduction of the whole-cell pertussis vaccine, the incidence of pertussis declined, reaching a nadir in the United States in 1976.^6^ However, due to concerns about reactions associated with whole-cell vaccine,^8–10^ an acellular vaccine was introduced in the 1990s,^6^ but this vaccine was less effective.^11^ Coinciding with the introduction of a less-effective vaccine, the number of pertussis cases reported in the United States has increased.^12–14^ Other reasons for the increase in cases include lower vaccination rates and the emergence of strains not included in the vaccine.^14^

Because pertussis is extremely contagious, it is critical to diagnose cases early to prevent further transmission. Delays in diagnosing pertussis have been associated with outbreaks, especially in healthcare settings.^15–17^ Early identification is important for 3 reasons. First, if patients can be identified early enough, treatment may ameliorate symptoms. Second, treatment within the first 3 weeks can prevent the spread of pertussis.^1^ Finally, earlier identification of cases (ie, without delay) can help identify individuals who have been exposed to pertussis to facilitate chemoprophylaxis.^1^ Unfortunately, efforts involving chemoprophylaxis and treatment are limited because substantial transmission has usually occurred prior to diagnosis.^18,19^ Adults and adolescents may be difficult to diagnose, especially if they were vaccinated as children because they may experience milder symptoms.^16^ Although delays in diagnosing pertussis, in the context of outbreaks, are commonly reported, the incidence, duration, and risk factors for delays remain unknown. In this study, we used a population-based approach to estimate the incidence and duration of diagnostic delays associated with pertussis and to describe potential risk factors associated with patients experiencing a diagnostic delay.

## Methods

### Data source

We used longitudinal insurance claims from the Merative MarketScan Commercial Claims and Encounters (CCAE) and Medicare Supplemental (MDCR) databases from 2001 through 2020, and Multi-State Medicaid databases from 2014 through 2018. Over this period, these databases contain claims for >233 million distinct enrollees, representing >6 billion enrollment months. Claims from outpatient (all ambulatory care not resulting in inpatient admission), emergency department (ED) and inpatient visits are provided for all enrollees. The analyses of these deidentified data was deemed non–human-subjects research by the University of Iowa Institutional Review Board.

### Study population

We identified all patients diagnosed with pertussis using the *International Classification of Disease, Ninth Revision Clinical Modification* (ICD-9-CM) diagnosis codes 033.X and ICD-10-CM codes A37.XX. We required continuous enrollment for at least 1 year prior to the pertussis diagnosis along with receipt of a prescription for azithromycin, clarithromycin, or erythromycin within 7 days of the diagnosis. The 1-year enrollment period was selected to establish a baseline level of expected healthcare utilization and ensure that the observed pertussis event represented the index diagnosis.

### Statistical analysis

We conducted 2 primary statistical analyses. First, we estimated the frequency and duration of diagnostic delays for pertussis by applying a bootstrapping approach previously utilized to identify missed diagnostic opportunities for infectious diseases in claims data.^20–22^ Second, we used a regression analysis to evaluate risk factors for experiencing a possible missed diagnostic opportunity. For both analyses, we start by identifying potential diagnostic delays by looking for symptomatically similar diagnoses (SSDs) that occurred during healthcare visits prior to the index pertussis diagnosis. We defined SSDs to be diagnoses that include, or share, similar symptoms to pertussis such as cough, fever, or exhaustion. Supplementary Table 1 lists all ICD-9 and ICD-10 codes used to identify SSD conditions. This type of retrospective approach has been commonly used to identify diagnostic delays associated with a variety of diseases.^23–27^

### Estimating incidence of diagnostic delays

To estimate the frequency and duration of diagnostic delays, we used a bootstrapping approach that employs a nested case-crossover design within the cohort of pertussis cases to account for coincidental healthcare utilization not attributable to diagnostic delays. This approach has been previously applied to study tuberculosis,^20^ herpes encephalitis,^21^ and histoplasmosis.^22^ Full details of this bootstrapping method are provided elsewhere.^28^ We started by counting the number of SSD-related visits each day during the year prior to the pertussis diagnosis. We then partitioned the year prior to diagnosis into a control window (when pertussis is assumed to be absent) and a case window that we refer to as the diagnostic opportunity window. We identified the crossover point using the CUSUM change-point detection method^29^ as the point when SSD-related visits significantly increase from baseline. We estimated the expected number of SSD visits by analyzing the trend in SSD visits during the control window, where pertussis is believed to be absent. We then extrapolated this trend forward to estimate the expected number of SSD visits during the diagnostic opportunity window. We computed the number of likely diagnostic opportunities as the excess number of SSD visits, computed as the difference between the observed and expected SSD visits during the diagnostic opportunity window. Finally, bootstrapping was used to select which individual visits represented “likely” missed opportunities. Specifically, given the estimated number of missed opportunities each day during the diagnostic opportunity window, we drew a corresponding number of patient visits and computed the number of patients experiencing a missed opportunity, number of missed opportunities per patient, and the duration of diagnostic delays. This procedure was repeated 25,000 times to compute the mean and 95% bootstrap-based confidence intervals around these estimates.

Because pertussis presentation and diagnosis may differ between children and adults, we also stratified results across different age groups. We segmented pertussis cases into the following age groups: <2, 2–4, 5–11, 12–17, and ≥18 years. For each age group, we then re-estimated the diagnostic opportunity window (ie, change point), the baseline level of SSD visits, computed the excess number of visits, and we reran the corresponding bootstrapping analysis.

### Estimating risk factors for experiencing a potential missed diagnostic opportunity

Given the estimated change point defining the diagnostic opportunity window (described above), we analyzed the risk factors for potential diagnostic delays by estimating the likelihood of a missed opportunity during healthcare visits prior to diagnosis. Specifically, we treated a missed diagnosis as a binary outcome and assigned a value of 1 (ie, missed opportunity) to SSD-related visits occurring on days during the diagnostic opportunity window and a 0 (ie, correct diagnosis) to the index diagnosis date. Unlike the bootstrapping methods, the risk factor model did not attempt to distinguish between coincidental visits and true missed opportunities; thus, we referred to these outcomes (SSD during diagnostic opportunity window) as potential missed opportunities. Because multiple visits occurring on a single day are likely to represent a linked episode of care, for each visit day observed during the diagnostic opportunity window we aggregated all SSD diagnoses recorded. We used a multivariate logistic regression analysis to estimate the likelihood of a visit day representing a missed opportunity while controlling for a range of potential risk factors.

We considered both patient- and context-specific risk factors as covariates in the model estimating the likelihood of a missed opportunity. Patient demographics included age, sex, urban-versus-rural location (only available in the CCAE and MDCR databases), and race (only available in the Medicaid database). Environment- and setting-specific factors included the year and month of the symptomatic visit-day or the index diagnosis, whether the visit day involved inpatient, outpatient, or ED settings, or combinations of multiple settings, and indicators for telehealth visits and a previous prescription for nonmacrolide antibiotics commonly associated with respiratory infections (see Table 1 notes for list of antibiotics).

**Table 1.** Baseline Characteristics of Final Study Cohort Using Marketscan Data

Characteristic	Patients, No. (%)		
	CCAE and MDCR	Medicaid	Overall
Total	18,898	1,930	20,828
**Age at diagnosis**			
<2 y	340 (1.8)	146 (7.6)	486 (2.3)
2–4 y	1,812 (9.6)	481 (24.9)	2,293 (11.0)
5–11y	4,031 (21.3)	677 (35.1)	4,708 (22.6)
12–17 y	3,416 (18.1)	273 (14.1)	3,689 (17.7)
18–35 y	2,638 (14.0)	196 (10.2)	2,834 (13.6)
36–45 y	2,159 (11.4)	80 (4.1)	2,239 (10.7)
46–55 y	2,107 (11.1)	44 (2.3)	2,151 (10.3)
56–65 y	1,729 (9.1)	29 (1.5)	1758 (8.4)
>65 y	666 (3.5)	4 (0.2)	670 (3.2)
Mean (SD)	26.6 (21.0)	12.0 (12.9)	25.3 (20.8)
Median (IQR)	17 (35.0)	8 (12.0)	16 (34.0)
**Sex**			
Male	7,855 (41.6)	869 (45.0)	8,724 (41.9)
Female	11,043 (58.4)	1,061 (55.0)	12,104 (58.1)
**Region**			
Rural	2,606 (13.8)	…	…
Urban	16,238 (85.9)	…	…
Missing	54 (0.3)	…	…
**Race**			
White	…	1,148 (59.5)	…
Black	…	318 (16.5)	…
Hispanic	…	152 (7.9)	…
Other	…	61 (3.2)	…
Missing	…	251 (13.0)	…
Receipt of respiratory antibiotic^a^ during diagnostic opportunity window^b^	1,720 (9.1)	179 (9.3)	1,899 (9.1)
Telehealth visit during diagnostic opportunity window^b^	38 (0.2)	0 (0.0)	38 (0.2)

Notes: CCAE, Commercial Claims and Encounters; MDCR, Medicare Supplemental; SD, standard deviation; IQR, interquartile range.

a Respiratory antibiotics included clindamycin, fluoroquinolones, cephalosporins, penicillins, and tetracyclines.

b The *diagnostic opportunity window* refers to the period of time where diagnostic opportunities may occur and was identified at 43 days prior to the pertussis diagnosis.

### Sensitivity analyses

Not all signs and symptoms present during a clinic visit may be captured by diagnostic codes in insurance claims, and the SSD visits identified may underestimate the true number of missed opportunities. Therefore, we repeated all our analyses to compute the frequency and duration of delays using all visits (with or without an SSD) as potential diagnostic opportunities. In addition, we performed our bootstrapping analyses separately for the CCAE/MDCR and Medicaid populations.

## Results

**Fig. 1.**
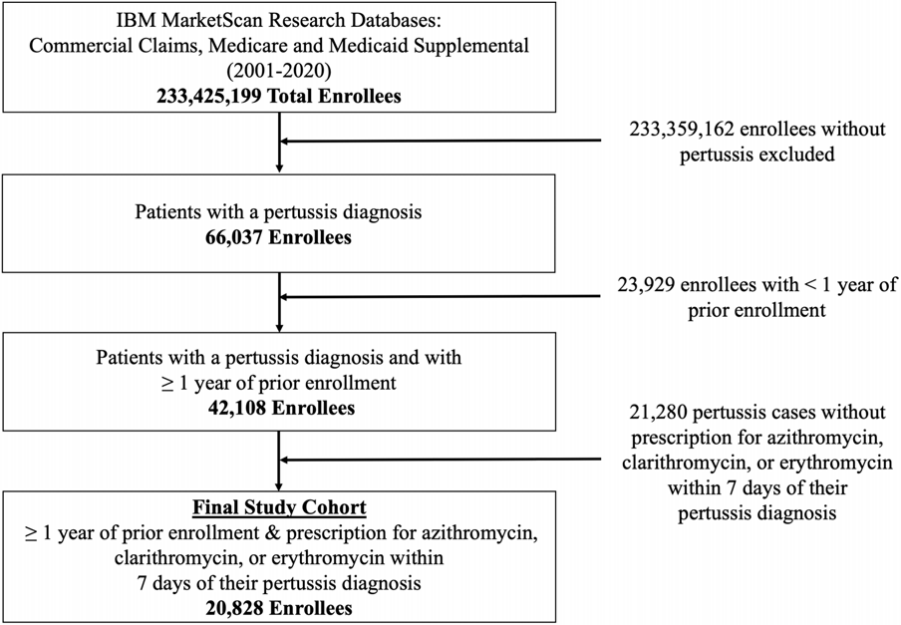
Flow diagram of patient inclusion and exclusion criteria. Counts of patients excluded and reasons for exclusion used to identify the final 20,828 index cases of pertussis.

From 2001 through 2020, a total of 66,037 individuals had a pertussis diagnosis. The final study cohort included 20,828 enrollees (Fig. 1) who were enrolled for at least 1 year prior to the pertussis diagnosis and received macrolide treatment within 7 days of the diagnosis. Table 1 summarizes baseline characteristics of the study population. Patients in the Medicaid cohort tended to be slightly younger with a slightly smaller proportion of female patients than those in the MDCR/CCAE cohort. The monthly age-adjusted rate of identified pertussis cases along with a comparison to annual CDC surveillance reports is provided in Supplementary Figures 1 and 2 (online). In general, trends in the incidence of identified pertussis cases matched surveillance estimates.

**Fig. 2.**
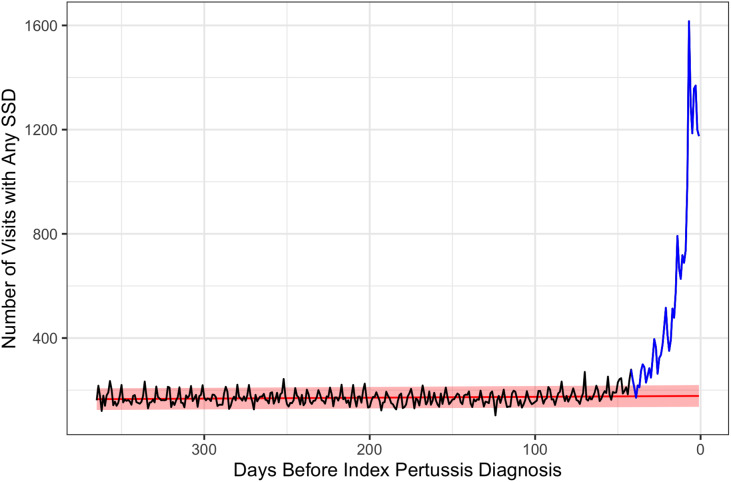
Trends in observed and expected number of SSD-related visits. The red line depicts the trend in expected SSD-related visits, which was estimated using data from the crossover control period prior to the change-point. The blue line depicts the trend in the observed number of visits during the diagnostic opportunity window (ie, after the change point.) The area between the blue and red lines depicts the number of SSD-related visits that represent likely diagnostic opportunities.

Figure 2 and Supplementary Figure 3 (online) depict the pattern of SSD-related visits that occurred in the year prior to the index pertussis diagnosis. A notable increase in the frequency of SSD-related visits occurred beginning ∼50 days before the index pertussis visit. Of the 20,828 case patients we identified, 20,229 (97.1%) patients had at least 1 healthcare visit (for any reason) in the year prior to their index pertussis diagnosis, and 15,960 (76.6%) patients had at least 1 SSD-related visit in the year prior to the pertussis diagnosis.

Because not all SSD visits may represent diagnostic opportunities, we used a bootstrapping approach to estimate the number of likely diagnostic opportunities based on the observed and expected number of SSD visits prior to the index pertussis diagnosis. The change-point analysis detected a significant increase in the number of SSD visits occurring 43 days (95% confidence interval [CI], 36–50) prior to the index diagnosis; this represents the crossover point used for our nested case-crossover analysis and start of the diagnostic-opportunity window. Figure 2 summarizes the observed and expected trend lines. Across all patients, 15,460 patients (74.2%) had at least 1 SSD during this diagnostic opportunity window. In total, 38,079 SSD visits occurred during the diagnostic opportunity window. Of these visits, we estimated that 14,413 (37.9%) represented a missed opportunity, and ∼81 (0.6%) occurred in inpatient settings, 12,139 (84.2%) occurred in outpatient settings, and 2,193 (15.2%) occurred in ED settings.

Table 2 provides individual estimates for the frequency and duration of diagnostic delays. An estimated 37.6% (95% CI, 36.6%–38.6%) of patients experienced at least one missed opportunity. Of patients who experienced a missed opportunity, we estimated that they experienced an average of 1.84 (95% CI, 1.80–1.88) visits representing a missed opportunity with a mean delay duration of 11.97 days (95% CI, 11.17–12.77). Because of the unitary nature of missed opportunities and delay duration, we performed a distributional breakdown of the estimated number of missed opportunities and delay duration (Table 2). For example, 17.4% (95% CI, 16.6%–18.2%) of patients experienced ≥2 missed opportunities and 1.6% (95% CI, 1.3%–1.8%) experienced 5 or more. Additionally, 57.2% (95% CI, 55.5%–58.7%) of patients had a delay duration of ≥8 days, and 6.7% (95% CI, 4.8%–9.1%) experienced delays of ≥28 days.

**Table 2.** Frequency of Missed Opportunities and Duration of Delay From Bootstrapping Results^a^

Metric/Category	Count of Patients (% of All Patients)^b^	95% CI (From Bootstrapping)
**No. of missed opportunities per patient**		
0 missed opportunities	13,003 (62.4)	12,798–13,211 (61.4–63.4)
≥1 missed opportunities	7,825 (37.6)	7,617–8,030 (36.6–38.6)
≥ 2 missed opportunities	3,618 (17.4)	3,455–3,786 (16.6–18.2)
≥3 missed opportunities	1,579 (7.6)	1,466–1,691 (7.0–8.1)
≥4 missed opportunities	718 (3.4)	645–7,91 (3.1–3.8)
≥5 missed opportunities	326 (1.6)	279–3,74 (1.3–1.8)
Mean number of missed opportunities per patient, overall^c^	1.84	1.80–1.88
Mean number of missed opportunities per patient, outpatient^c^	1.55	1.52–1.59
Mean number of missed opportunities per patient, inpatient^c^	0.01	0.01–0.01
Mean number of missed opportunities per patient, ED^c^	0.28	0.27–0.30
**Duration of delays**		
≥ 1 d	7,825 (100.0)	7,617–8,030 (NA)
≥4 d	6,613 (84.3)	6,432–6,814 (83.4–85.3)
≥8 d	4,466 (57.2)	4,278–4,671 (55.5–58.7)
≥12 d	3,262 (41.8)	3,075–3,452 (39.8–43.7)
≥16 d	2,161 (27.7)	1,990–2,348 (25.8–29.6)
≥20 d	1,398 (17.9)	1,196–1,590 (15.5–20.2)
≥24 d	980 (12.5)	687–1,212 (9.0–15.2)
≥ 28 d	514 (6.7)	361–703 (4.8–9.1)
Mean delay duration, d	11.97	11.17–12.77
Median delay duration, d	10	9–10

a Units in counts (or % of all patients) unless otherwise specified.

b The distribution and mean number of missed opportunities each patient experienced along with the distribution and mean and median duration (in days) of diagnostic delays are presented. Missed opportunities represent healthcare visits in which symptoms were present, but pertussis was not diagnosed. Delay duration was defined as the time between the earliest missed opportunity a patient experienced and their index diagnosis.

c Mean and medians correspond to the number of missed opportunities per patient.

**Fig. 3.**
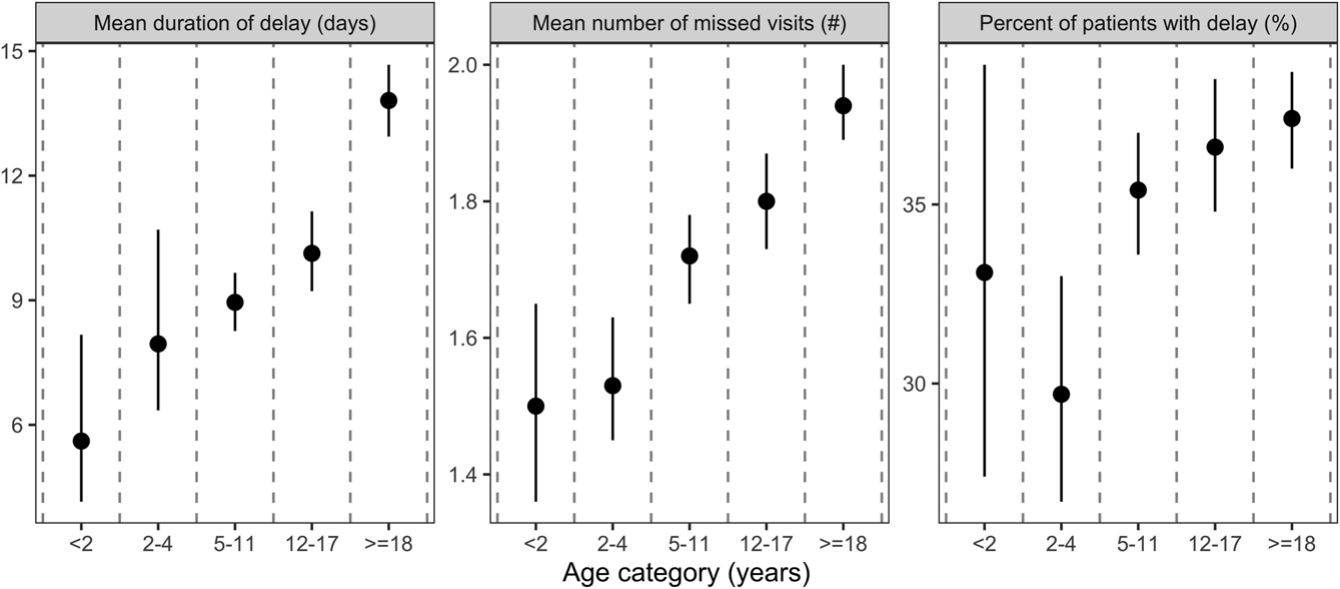
Results for duration, number of missed opportunities, and percentage of patients with a delay for different age groups.

Table 3 and Figure 3 present results of the bootstrapping analysis when stratified by different age groups. Two primary findings can be observed when comparing results across ages. First, adults were slightly more likely to experience a missed opportunity. The percentage of patients experiencing at least 1 missed opportunity ranged from 29.7% (95% CI, 26.7%–33.0%) for those aged 2–4 years to 37.4% (95% CI, 36.0%–38.7%) for those aged ≥18 years. Similarly, the mean number of missed opportunities per patient ranged from 1.50 (95% CI, 1.36–1.65) for those aged <2 years to 1.94 (95% CI, 1.80–1.88) for those aged ≥18. Second, the duration of delays tended to monotonically increase across age groups, and the duration more than doubled for adults compared to the youngest age group. The mean duration of delays increased from 5.61 days (95% CI, 4.15–8.17) for patients aged <2 years to 13.81 days (95% CI, 12.94–14.67) for patients aged ≥18 years.

**Table 3.** Age-Stratified Results From Bootstrapping Analysis for Number of Missed Opportunities and Delay Duration

Variable	Age Group					
	<2 Years	2–4 Years	5–11 Years	12–17 Years	≥18 Years	All Ages
Estimated change point	35	29	36	43	50	43
No. of patients (% of all patients)	486 (2.3)	2,293 (11.0)	4,708 (22.6)	3,689 (17.7)	9,652 (46.3)	20,828 (100)
No. of missed opportunities (95% CI)	243 (190–297)	1,048 (904–1,230)	2,864 (2,653–3,050)	2,431 (2,261–2,614)	7,014 (6,650–7,383)	14,413 (13,827–15,019)
% of patients with at least 1 missed opportunity (95% CI)	33.1 (27.4–38.9)	29.7 (26.7–33.0)	35.4 (33.6–37.0)	36.6 (34.8–38.5)	37.4 (36.0–38.7)	37.6 (36.6–38.6)
Mean no. of missed opportunities per patient (95% CI)	1.50 (1.36–1.65)	1.53 (1.45–1.63)	1.72 (1.65–1.78)	1.80 (1.73–1.87)	1.94 (1.89–2.00)	1.84 (1.80–1.88)
Mean duration of delays (95% CI)	5.61 (4.15–8.17)	7.95 (6.35–10.70)	8.95 (8.26–9.66)	10.13 (9.22–11.14)	13.81 (12.94–14.67)	11.97 (11.17–12.77)

Note: Distributional metrics for the number of patients with different values of the number missed opportunities and duration of delays are provided in Supplementary Table 2 (online).

We conducted 2 sensitivity analyses. First, to provide an upper bound of potential missed opportunities not captured by claims or our SSD list, we repeated our analysis using all visits regardless of the presence of an SSD. Supplementary Table 3 (online) lists these findings which resulted in a slightly greater frequency and duration of diagnostic delays. We estimated that 46.4% (95% CI, 45.2%–47.7%) of patients experienced a missed opportunity, with an average of 2.03 (95% CI, 1.99–2.09) missed opportunities per patient, and a delay duration of 11.32 days (95% CI, 10.57–12.21). Second, we considered analyses stratified by the CCAE and MDCR databases versus the Medicaid database (Supplementary Table 4 online). In general, the frequency and duration estimates were similar when the databases were stratified. The frequency and duration appear slightly lower in the Medicaid cohort, but this result is attributable to the younger Medicaid population.

Table 4 presents results of the logistic regression model estimating the likelihood of experiencing a potential missed opportunity during a visit on a given day. Several context- and patient-level factors were associated with increased likelihood of potential missed opportunities. Visiting the ED without an associated outpatient or inpatient stay on the same day was associated with an increased likelihood of delay. Potential delays were more likely to occur during weekend visits. Patients who received antibiotics commonly used to treat respiratory infections during the diagnostic-opportunity window were more likely to experience a potential delayed diagnosis for pertussis. Additionally, we observed that patients who had telehealth visits during the diagnostic opportunity window were more likely to experience a potential delayed diagnosis for pertussis.

**Table 4.** Multivariate Regression Results for Likelihood of Experiencing a Delay

Coefficient	CCAE and MDCR		Medicaid	
	Effect Estimate (95% CI)	*P* Value	Effect Estimate (95% CI)	*P* Value
Weekend (visits that occurred on a Saturday or Sunday)	1.171 (1.090–1.258)	<.001	1.104 (0.890–1.370)	.368
Sex, female	1.029 (0.985–1.075)	.199	0.877 (0.761–1.011)	.069
**Age group**				
<2 y	REF	REF	REF	REF
2–4 y	0.760 (0.646–0.895)	.001	0.873 (0.641–1.190)	.391
5–11 y	0.765 (0.655–0.894)	<.001	0.672 (0.498–0.907)	.009
12–17 y	0.721 (0.616–0.843)	<.001	0.687 (0.494–0.954)	.025
18–35 y	0.601 (0.512–0.706)	<.001	0.679 (0.475–0.971)	.034
36–45 y	0.705 (0.600–0.829)	<.001	0.958 (0.630–1.456)	.840
46–55 y	0.781 (0.665–0.918)	.003	0.494 (0.276–0.884)	.017
56–65 y	0.887 (0.753–1.044)	.148	0.884 (0.478–1.634)	.693
>65 y	1.077 (0.898–1.291)	.424	1.144 (0.283–4.620)	.850
**Settings visited on a given day** ^**a**^				
Outpatient only	REF	REF	REF	REF
All 3 (inpatient, outpatient, and ED)	0.205 (0.119–0.353)	<.001	1.034 (0.063–16.843)	.981
ED only	1.473 (1.316–1.649)	<.001	0.964 (0.798–1.164)	.702
Inpatient only	0.287 (0.213–0.388)	<.001	0.170 (0.063–0.457)	<.001
Inpatient and ED	0.116 (0.034–0.399)	<.001	…	…
Inpatient and outpatient	0.330 (0.241–0.451)	<.001	0.246 (0.077–0.779)	.017
Outpatient and ED	1.496 (1.350–1.657)	<.001	0.933 (0.717–1.214)	.605
Respiratory antibiotics between change point and 1 d prior to index	3.091 (2.903–3.292)	<.001	2.780 (2.269–3.407)	<.001
Telehealth visits between change point and 1 d prior to index	1.650 (1.083–2.515)	.020	…	…
Urban vs not urban^b^	0.957 (0.900–1.017)	.160	…	…
**Race** ^**c**^				
White	…	…	REF	REF
Black	…	…	0.929 (0.775–1.114)	.427
Hispanic	…	…	0.924 (0.721–1.183)	.530
Other	…	…	0.791 (0.534–1.170)	.240

Notes: ED, emergency department; CCAE, Commercial Claims and Encounters; MDCR, Medicare Supplemental. The model was also adjusted for year and month of SSD/index visit.

a Visits were aggregated to a daily level, and the different combination of healthcare settings visited on a given day were identified.

b Patient location information is not available in the Medicaid database.

c Patient race is not available in the CCAE/MDCR databases.

## Discussion

Our results demonstrate that patients with pertussis often experience diagnostic delays. For example, 18% of pertussis patients had a delay of at least 20 days, and the average delay was 12 days. These delays have important implications for patient health because, if caught quickly, pertussis is treatable, and early treatment can also prevent transmission of the disease. On average, patients had almost 2 visits prior to diagnosis, and some had many more visits. At each of these visits, multiple healthcare professionals and other patients were potentially exposed to pertussis, and between these visits, many community and household exposures undoubtably occurred.

The frequency and duration of diagnostic delays varied across age groups. In general, the percentage of patients experiencing a delay tended to increase with age from 2–4 years up to adulthood (≥18 years). Both the number of missed opportunities per patient and the duration of delays increased monotonically from age <2 years through adulthood. Thus, diagnostic delays for pertussis among adults appear to be both longer and more frequent compared to younger children. Pertussis has long been viewed as a childhood illness.^16^ Thus, it is not surprising that older individuals are more likely to experience a diagnostic delay. Although most hospitalizations and deaths occur among infants,^30–32^ in the postvaccination era, cases commonly occur among adolescents and adults. Indeed, diagnosing pertussis in adults is more difficult because these cases may present with mild cough and other respiratory symptoms.^16^ This is further complicated because cough is a leading cause of outpatient visits.^33^ Up to 17% of adolescents and adults with a prolonged history of cough (eg, >2 weeks) have pertussis.^34^ Thus, pertussis should be part of the differential diagnosis for patients with prolonged cough. While patients with symptoms longer than a few weeks may no longer benefit from treatment themselves, identification of these cases will help the public health response. Specifically, more timely diagnosis can facilitate contact tracing, help minimize contacts, and inform the administration of chemoprophylaxis among exposures.

We identified 2 other potential risk factors for diagnostic delays associated with pertussis. First, prior receipt of an antibiotic commonly used to treat respiratory infections was associated with a diagnostic delay. We did not include macrolides because these drugs were used to identify pertussis cases. Assuming that these potential therapeutic interventions would be successful likely results in delayed diagnostic testing (eg, patients told to wait for these treatments to take effect). Second, diagnostic delays were also more common for patients who visited the ED, without an inpatient visit on the same day. Diagnostic errors may occur commonly in the ED setting, and revisits to an ED are often due to misdiagnosis.^35^ In the ED, physicians are often treating patients they are seeing for the first time, and they may be unaware of the patients’ medical history. In addition, many patients have vague symptoms with a wide range of severity,^36^ and physicians in the ED are often interrupted.^37^ Finally, when diagnostic errors do occur, ED physicians may not be able to learn from missed opportunities to diagnose a disease because follow-up care occurs in other healthcare settings. Improving clinical suspicion for pertussis is clearly needed. Improving illness scripts among clinicians could improve diagnostic accuracy. Future work describing different clinical presentations in different clinical settings may also help strategies to decrease diagnostic delays.

Our study had several limitations. First, we relied on diagnostic codes from claims to identify pertussis cases and potential symptoms prior to diagnosis. However, we also used outpatient medications to validate our case definition, and we conducted sensitivity analyses using all visits. We only captured patients who were eventually diagnosed; thus, our findings may represent underestimates. Second, our data set was restricted to an insured population, with employer-sponsored health insurance or privately managed Medicaid coverage, and our findings may not be generalizable to uninsured populations. Third, vaccination may make it more difficult to diagnose people with pertussis, but we were not able to account for vaccination in our investigation. Finally, we grouped all adults together, but there may be specific risk factors for older adults, and these should be addressed in future work.

Despite an effective vaccine, pertussis has been reemerging both worldwide and in the United States. Given the limitations of vaccination strategies and approaches, the early identification of cases may play an important role in the public health response. However, our results show that diagnostic delays are frequent, and more work is needed to improve timely diagnostic strategies, especially among adolescents and adults.

## Supplementary material

For supplementary material accompanying this paper visit https://doi.org/10.1017/ice.2023.31. click here to view supplementary material
