# Supplementary material for: The incidence, duration, risk factors, and age-based variation of missed opportunities to diagnose pertussis: A population-based cohort study
Source: Infect Control Hosp Epidemiol. 2023 Mar 15;44(10):1629–36. doi: 10.1017/ice.2023.31 (PMC10587384; doi:10.1017/ice.2023.31)

**Supplementary Material**

**Supplementary Tables**

**Supplementary Table 1 - List of diagnosis codes for SSDs used to identify potential diagnostic opportunities**

| **Category** | **ICD-9-CM** | **ICD-10-CM** |
| --- | --- | --- |
| Symptom-based diagnoses | 388.70, 536.2, 780.2, 780.6, 780.60, 780.79, 784.1, 784.42, 784.99, 785.9, 786.05, 786.07, 786.09, 786.1, 786.2, 786.30, 786.50, 786.51, 786.52, 786.59, 786.9, 787.01, 787.03, 799.3, 799.83 | R04.2, R05, R06.00, R06.02, R06.03, R06.09, R06.1, R06.2, R06.3, R06.83, R06.89, R07.0, R07.1, R07.2, R07.81, R07.82, R07.89, R07.9, R09.81, R09.82, R09.89, R11.2, R11.10, R11.11, R11.12, R49.0, R50.9, R53.1, R53.81, R53.83, R55, R68.13 |
| Alternative Infectious Disease-based diagnoses | 034.0, 079.6, 079.89, 079.99, 381.00, 381.01, 382.00, 382.9, 460, 461.0, 461.1, 461.8, 461.9, 462, 463, 464.00, 464.01, 464.21, 464.4, 465.8, 465.9, 466.0, 466.11, 466.19, 473.0, 473.8, 473.9, 477.8, 477.9, 481, 482.9, 485, 486, 487.1, 488.12, 490, 491.20, 491.21, 790.8 | B34.8, B34.9, B97.4, B97.89, H65.199, H66.009, H66.90, J00, J01.00, J01.10, J01.40, J01.80, J01.90, J02.0, J02.8, J02.9, J03.90, J04.0, J05.0, J06.9, J10.1, J11.1, J15.9, J18.0, J18.1, J18.8, J18.9, J20.8, J20.9, J21.0, J21.8, J21.9, J32.0, J32.4, J32.8, J32.9, J40, J42 |
| Alternative-Sino-Pulmonary-Esophageal-based diagnoses | 381.00, 382.00, 382.9, 472.0, 477.9, 478.19, 491.21, 491.9, 493.00, 493.02, 493.10, 493.12, 493.20, 493.22, 493.82, 493.90, 493.91, 493.92, 496, 507.0, 507.8, 514, 515, 518.0, 518.81, 518.82, 518.89, 519.11, 519.8, 519.9, 530.11, 530.81, 784.91, 786.09, 793.11 | H65.02, H92.03, I27.0, I27.29, J30.0, J30.2, J30.89, J30.9, J31.0, J44.1, J44.9, J45.20, J45.21, J45.30, J45.31, J45.40, J45.41, J45.901, J45.902, J45.909, J45.991, J45.998, J69.0, J69.8, J80, J81.1, J96.00, J96.90, J98.01, J98.4, J98.8, J98.9, K21.0, K21.9, R09.82, R91.1 |
| Testing Imaging or Physical Exam-based diagnoses | 288.60, 288.61, 288.69, 511.1, 511.89, 518.0, 518.51, 518.81, 782.5, 786.03, 786.7, 793.1, 793.11, 793.19, 799.02 | D72.820, D72.828, D72.829, J90, J96.01, J96.00, J96.90, J98.11, J98.19, R06.81, R09.02, R23.0, R91.8, V74.1 |

**Supplementary Table 2 -** Distributional metrics for age-stratified bootstrapping analysis corresponding to the results in Table 3.

|  | **Age Group** | | | | | |
| --- | --- | --- | --- | --- | --- | --- |
|  | $\boldsymbol{<}$**2 years**  Count (% of patients)  [95% CI from bootstrapping] | **2-4 years**  Count (% of patients)  [95% CI from bootstrapping] | **5-11 years**  Count (% of patients)  [95% CI from bootstrapping] | **12-17 years**  Count (% of patients)  [95% CI from bootstrapping] | $\boldsymbol{\geq}$**18 years**  Count (% of patients)  [95% CI from bootstrapping] | **All Ages**  Count (% of patients)  [95% CI from bootstrapping] |
| ***Number of missed opportunities per patient*** | | | | | | |
| 0 missed opportunities | 325 (66.9%) [297 - 353 (61.1 - 72.6%)] | 1611 (70.3%) [1537 - 1680 (67.0 - 73.3%)] | 3039 (64.6%) [2965 - 3125 (63.0 - 66.4%)] | 2338 (63.4%) [2270 - 2404 (61.5 - 65.2%)] | 6046 (62.6%) [5919 - 6173 (61.3 - 64.0%)] | 13003 (62.4%) [12798 - 13211 (61.4 - 63.4%)] |
| ≥ 1 missed opportunity | 161 (33.1%) [133 - 189 (27.4 - 38.9%)] | 682 (29.7%) [613 - 756 (26.7 - 33.0%)] | 1669 (35.4%) [1583 - 1743 (33.6 - 37.0%)] | 1351 (36.6%) [1285 - 1419 (34.8 - 38.5%)] | 3606 (37.4%) [3479 - 3733 (36.0 - 38.7%)] | 7825 (37.6%) [7617 - 8030 (36.6 - 38.6%)] |
| ≥ 2 missed opportunities | 58 (11.8%) [40 - 77 (8.2 - 15.8%)] | 239 (10.4%) [199 - 287 (8.7 - 12.5%)] | 714 (15.2%) [653 - 771 (13.9 - 16.4%)] | 629 (17.0%) [578 - 681 (15.7 - 18.5%)] | 1749 (18.1%) [1647 - 1851 (17.1 - 19.2%)] | 3618 (17.4%) [3455 - 3786 (16.6 - 18.2%)] |
| ≥ 3 missed opportunities | 16 (3.3%) [8 - 26 (1.6 - 5.3%)] | 76 (3.3%) [54 - 102 (2.4 - 4.4%)] | 273 (5.8%) [234 - 313 (5.0 - 6.6%)] | 254 (6.9%) [217 - 291 (5.9 - 7.9%)] | 834 (8.6%) [762 - 907 (7.9 - 9.4%)] | 1579 (7.6%) [1466 - 1691 (7.0 - 8.1%)] |
| ≥ 4 missed opportunities | 5 (1.1%) [1 - 11 (0.2 - 2.3%)] | 25 (1.1%) [14 - 39 (0.6 - 1.7%)] | 113 (2.4%) [89 - 138 (1.9 - 2.9%)] | 110 (3.0%) [86 - 134 (2.3 - 3.6%)] | 405 (4.2%) [355 - 456 (3.7 - 4.7%)] | 718 (3.4%) [645 - 791 (3.1 - 3.8%)] |
| ≥ 5 missed opportunities | 2 (0.4%) [0 - 5 (0.0 - 1.0%)] | 10 (0.4%) [4 - 18 (0.2 - 0.8%)] | 49 (1.1%) [35 - 65 (0.7 - 1.4%)] | 45 (1.2%) [31 - 60 (0.8 - 1.6%)] | 198 (2.1%) [165 - 233 (1.7 - 2.4%)] | 326 (1.6%) [279 - 374 (1.3 - 1.8%)] |
| ***Duration of delays (days)*** | | | | | | |
| ≥ 1 Day | 161 (100.0%) [133 - 189 (NA)] | 682 (100.0%) [613 - 756 (NA)] | 1669 (100.0%) [1583 - 1743 (NA)] | 1351 (100.0%) [1285 - 1419 (NA)] | 3606 (100.0%) [3479 - 3733 (NA)] | 7825 (100.0%) [7617 - 8030 (NA)] |
| ≥ 4 Days | 98 (60.9%) [72 - 124 (49.0 - 71.3%)] | 491 (70.3%) [416 - 560 (63.9 - 75.4%)] | 1323 (78.1%) [1265 - 1392 (76.3 - 79.9%)] | 1131 (83.6%) [1061 - 1200 (81.4 - 85.8%)] | 3199 (88.8%) [3071 - 3331 (87.6 - 90.0%)] | 6613 (84.3%) [6432 - 6814 (83.4 - 85.3%)] |
| ≥ 8 Days | 29 (17.8%) [10 - 47 (6.7 - 27.2%)] | 240 (35.6%) [180 - 316 (28.7 - 43.1%)] | 747 (45.0%) [672 - 821 (41.5 - 48.2%)] | 688 (50.9%) [617 - 753 (47.2 - 54.3%)] | 2296 (64.2%) [2182 - 2441 (62.1 - 66.5%)] | 4466 (57.2%) [4278 - 4671 (55.5 - 58.7%)] |
| ≥ 12 Days | 16 (10.1%) [2 - 35 (1.3 - 20.6%)] | 137 (20.1%) [81 - 202 (12.7 - 27.4%)] | 483 (28.9%) [407 - 553 (25.4 - 32.5%)] | 458 (33.9%) [400 - 518 (30.3 - 37.5%)] | 1759 (49.2%) [1642 - 1904 (46.5 - 51.8%)] | 3262 (41.8%) [3075 - 3452 (39.8 - 43.7%)] |
| ≥ 16 Days | 5 (3.0%) [0 - 16 (0.0 - 9.7%)] | 61 (8.8%) [17 - 129 (2.6 - 17.4%)] | 260 (15.6%) [199 - 337 (12.0 - 19.6%)] | 259 (19.2%) [201 - 314 (15.1 - 22.6%)] | 1230 (34.2%) [1091 - 1392 (30.7 - 37.8%)] | 2161 (27.7%) [1990 - 2348 (25.8 - 29.6%)] |
| ≥ 20 Days | 4 (2.5%) [0 - 12 (0.0 - 7.6%)] | 43 (6.3%) [10 - 95 (1.6 - 13.4%) | 152 (9.1%) [105 - 203 (6.3 - 12.2%)] | 176 (12.9%) [102 - 243 (7.7 - 17.0%)] | 931 (25.9%) [822 - 1069 (23.0 - 29.2%)] | 1398 (17.9%) [1196 - 1590 (15.5 - 20.2%)] |
| ≥ 24 Days | - | 26 (3.9%) [4 - 63 (0.7 - 8.4%)] | 65 (3.9%) [27 - 107 (1.6 - 6.3%)] | 83 (6.1%) [35 - 131 (2.6 - 9.6%)] | 611 (16.9%) [499 - 719 (14.1 - 19.7%)] | 980 (12.5%) [687 - 1212 (9.0 - 15.2%)] |
| ≥ 28 Days | - | 11 (1.6%) [0 - 50 (0.0 - 7.3%)] | 40 (2.4%) [12 - 77 (0.7 - 4.5%)] | 37 (2.8%) [6 - 69 (0.5 - 5.1%)] | 428 (11.8%) [320 - 515 (9.1 - 14.1%)] | 514 (6.7%) [61 - 703 (4.8 – 9.1%)] |

**Supplementary Table 3** – Sensitivity analysis - bootstrapping results using all visits (regardless of the presence of an SSD). Note: for these sensitivity results the estimated diagnostic-opportunity window began 50 (CI: 42-57) days prior to diagnosis. A total of 90,694 visits for any reason from 16,158 (77.6%) patients occurred during the diagnostic-opportunity window, of these visits, 19,662 (21.7%) were estimated to be missed opportunities.

| **Metric/Category** | **Count of patients (percentage of all patients) or Mean/Median per patient** | **95% CI (from bootstrapping)** |
| --- | --- | --- |
| ***Number of missed opportunities*** ***per patient*** | | |
| 0 missed opportunities | 11164 (53.6%) | 10895 - 11420 (52.3 - 54.8%) |
| ≥ 1 missed opportunity | 9664 (46.4%) | 9408 - 9933 (45.2 - 47.7%) |
| ≥ 2 missed opportunities | 5125 (24.6%) | 4885 - 5384 (23.5 - 25.8%) |
| ≥ 3 missed opportunities | 2518 (12.1%) | 2342 - 2715 (11.2 - 13.0%) |
| ≥ 4 missed opportunities | 1194 (5.7%) | 1079 - 1322 (5.2 - 6.3%) |
| ≥ 5 missed opportunities | 562 (2.7%) | 491 - 642 (2.4 - 3.1%) |
| Mean* number of missed opportunities per patient - Overall | 2.03 | 1.99 - 2.09 |
| Mean* number of missed opportunities per patient - Outpatient | 1.89 | 1.85 - 1.94 |
| Mean* number of missed opportunities per patient - Inpatient | 0.00 | 0.00 - 0.01 |
| Mean* number of missed opportunities per patient - Emergency Department | 0.14 | 0.13 - 0.15 |
| ***Duration of delays (days)*** | | |
| ≥ 1 Day | 9664 (100.0%) | 9408 - 9933 (100.0 - 100.0%) |
| ≥ 4 Days | 8125 (84.1%) | 7850 - 8400 (83.0 - 85.3%) |
| ≥ 8 Days | 5285 (54.9%) | 4997 - 5579 (52.8 - 56.9%) |
| ≥ 12 Days | 3803 (39.5%) | 3531 - 4096 (37.1 - 41.8%) |
| ≥ 16 Days | 3212 (33.2%) | 2869 - 3515 (30.3 - 35.7%) |
| ≥ 20 Days | 2217 (23.0%) | 1877 - 2544 (19.9 - 26.0%) |
| ≥ 24 Days | 1808 (18.7%) | 1526 - 2113 (16.1 - 21.5%) |
| ≥ 28 Days | 1498 (15.6%) | 1258 - 1728 (13.2 - 17.8%) |
| Mean Delay Duration (days) | 11.32 | 10.57 - 12.21 |
| Median Delay Duration (days) | 9 | 8 - 9 |

* Mean/Medians correspond to the number of missed opportunities per patient

**Supplementary Table 4** – Bootstrapping results stratified by Medicaid and CCAE or MDCR database.

| **Metric/Category** | **MDCR/CCAE** | | **MEDICAID** | | **OVERALL** | |
| --- | --- | --- | --- | --- | --- | --- |
|  | **Count of patients (percentage of all patients)/Mean** | **95% CI (from bootstrapping)** | **Count of patients (percentage of all patients)/Mean** | **95% CI (from bootstrapping)** | **Count of patients (percentage of all patients)/Mean** | **95% CI (from bootstrapping)** |
| ***Number of missed opportunities*** ***per patient*** | | | | | | |
| 0 missed opportunities | 11880 (62.9%) | 11683 - 12080 (61.8 - 63.9%) | 1201 (62.2%) | 1136 - 1260 (58.9 - 65.3%) | 13003 (62.4%) | 12798 - 13211 (61.4 - 63.4%) |
| ≥ 1 missed opportunity | 7018 (37.1%) | 6818 - 7215 (36.1 - 38.2%) | 729 (37.8%) | 670 - 794 (34.7 - 41.1%) | 7825 (37.6%) | 7617 - 8030 (36.6 - 38.6%) |
| ≥ 2 missed opportunities | 3237 (17.1%) | 3086 - 3387 (16.3 - 17.9%) | 322 (16.7%) | 277 - 374 (14.4 - 19.4%) | 3618 (17.4%) | 3455 - 3786 (16.6 - 18.2%) |
| ≥ 3 missed opportunities | 1430 (7.6%) | 1330 - 1538 (7.0 - 8.1%) | 117 (6.0%) | 90 - 147 (4.7 - 7.6%) | 1579 (7.6%) | 1466 - 1691 (7.0 - 8.1%) |
| ≥ 4 missed opportunities | 655 (3.5%) | 590 - 725 (3.1 - 3.8%) | 48 (2.5%) | 32 - 65 (1.7 - 3.4%) | 718 (3.4%) | 645 - 791 (3.1 - 3.8%) |
| ≥ 5 missed opportunities | 298 (1.6%) | 257 - 343 (1.4 - 1.8%) | 22 (1.1%) | 12 - 33 (0.6 - 1.7%) | 326 (1.6%) | 279 - 374 (1.3 - 1.8%) |
| Mean* - Overall | 1.84 | 1.80 - 1.88 | 1.74 | 1.64 - 1.86 | 1.84 | 1.80 - 1.88 |
| Mean* - Outpatient | 1.58 | 1.54 - 1.61 | 1.25 | 1.15 - 1.36 | 1.55 | 1.52 - 1.59 |
| Mean* - Inpatient | 0.01 | 0.01 - 0.01 | 0.01 | 0.00 - 0.02 | 0.01 | 0.01 - 0.01 |
| Mean* - Emergency Department | 0.25 | 0.24 - 0.27 | 0.48 | 0.43 - 0.54 | 0.28 | 0.27 - 0.30 |
| ***Duration of delays (days)*** | | | | | | |
| ≥ 1 Day | 7018 (100.0%) | 6818 - 7215 (100.0 - 100.0%) | 729 (100.0%) | 670 - 794 (100.0 - 100.0%) | 7825 (100.0%) | 7617 - 8030 (NA) |
| ≥ 4 Days | 5967 (84.8%) | 5779 - 6160 (83.9 - 85.9%) | 562 (76.7%) | 509 - 638 (72.9 - 80.6%) | 6613 (84.3%) | 6432 - 6814 (83.4 - 85.3%) |
| ≥ 8 Days | 4066 (58.0%) | 3902 - 4232 (56.6 - 59.7%) | 283 (39.4%) | 228 - 354 (33.4 - 45.8%) | 4466 (57.2%) | 4278 - 4671 (55.5 - 58.7%) |
| ≥ 12 Days | 2981 (42.6%) | 2813 - 3138 (40.8 - 44.3%) | 225 (30.7%) | 159 - 294 (23.0 - 38.0%) | 3262 (41.8%) | 3075 - 3452 (39.8 - 43.7%) |
| ≥ 16 Days | 1975 (28.2%) | 1819 - 2120 (26.3 - 30.0%) | 154 (21.2%) | 108 - 214 (15.6 - 27.9%) | 2161 (27.7%) | 1990 - 2348 (25.8 - 29.6%) |
| ≥ 20 Days | 1636 (23.1%) | 1491 - 1794 (21.2 - 24.9%) | 146 (17.3%) | 97 - 220 (11.8 - 24.7%) | 1398 (17.9%) | 1196 - 1590 (15.5 - 20.2%) |
| ≥ 24 Days | 923 (13.1%) | 690 - 1096 (10.0 - 15.3%) | 138 (18.6%) | 86 - 184 (12.2 - 24.0%) | 980 (12.5%) | 687 - 1212 (9.0 - 15.2%) |
| ≥ 28 Days | 643 (8.5%) | 391 - 883 (5.2 - 11.5%) | 124 (14.3%) | 53 - 259 (6.3 - 28.4%) | 514 (6.7%) | 361 - 703 (4.8 - 9.1%) |
| Mean Duration | 12.15 | 11.37 - 12.93 | 9.00 | 7.29 - 11.31 | 11.97 | 11.17 - 12.77 |
| Median Duration | 10 | 9 - 10 | 7 | 6 - 7 | 10 | 9 - 10 |

* Mean/Medians correspond to the number of missed opportunities per patient

**Supplementary Figures**

**Supplementary Figure 1 –** Age-adjusted monthly incidence of pertussis cases identified in the MarketScan claims data using the final cohort of identified controls.

**
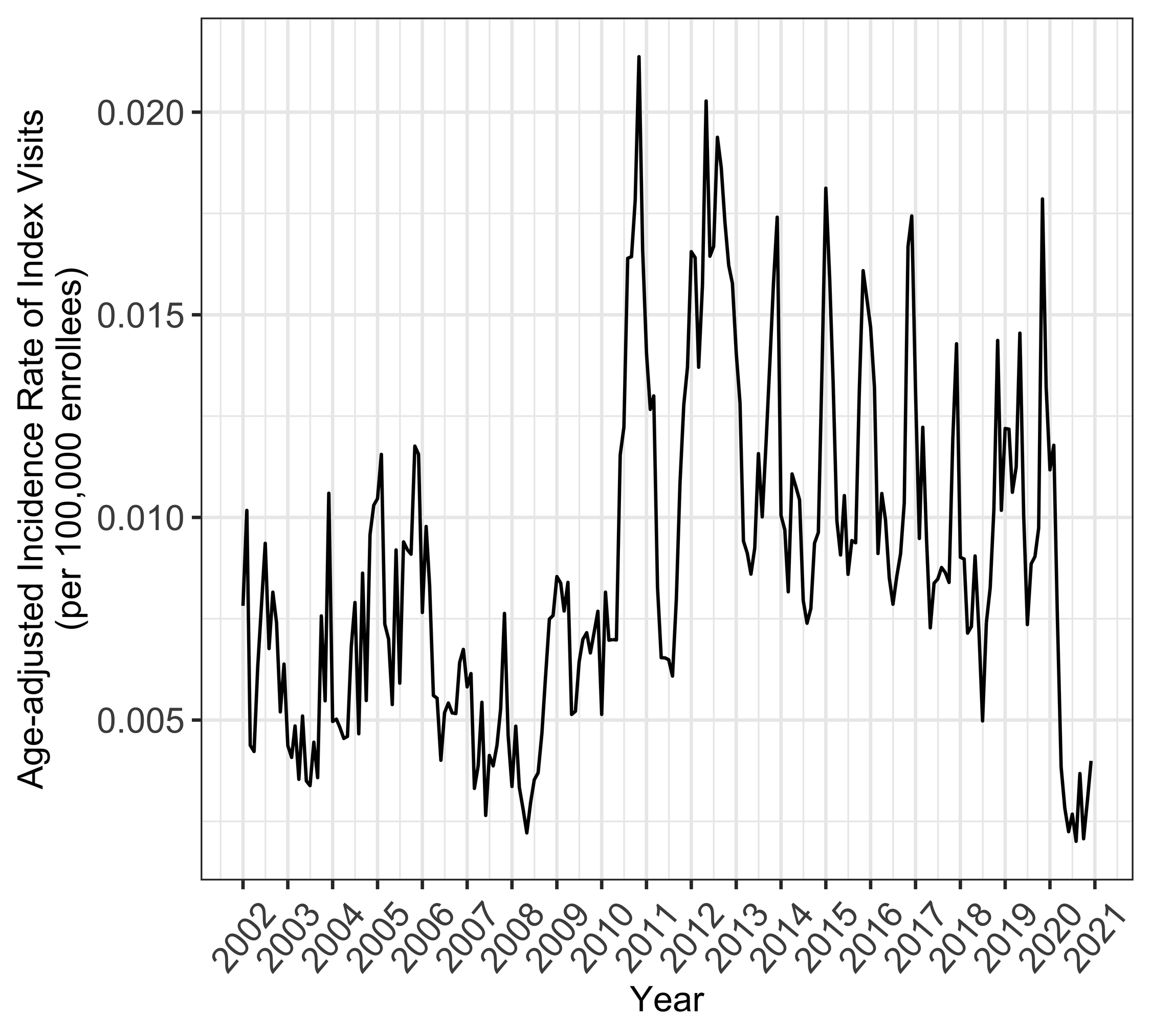
**

**Supplementary Figure 2 –** Comparison in trends in the annual incidence of identified pertussis cases using MarketScan claims versus CDC reported cases (CDC data reported at <https://www.cdc.gov/pertussis/surv-reporting/cases-by-year.html>). Incidence from all 66,037 identified pertussis cases is depicted in red, with the 20,828 pertussis cases in the final cohort depicted in blue, and the CDC reported incidence is depicted in green. Note: claims-based incidence was computed as the total number of identified cases divided by the average total annual enrollment. CDC surveillance incidence is computed as the total number of annual cases divided by US Census population estimates.

**Supplementary Figure 3 –** Trends in SSD visits and all visits prior to the index

pertussis diagnosis by type of healthcare setting. The red lines depict

all visits, and the blue line depicts visits with SSD-related conditions


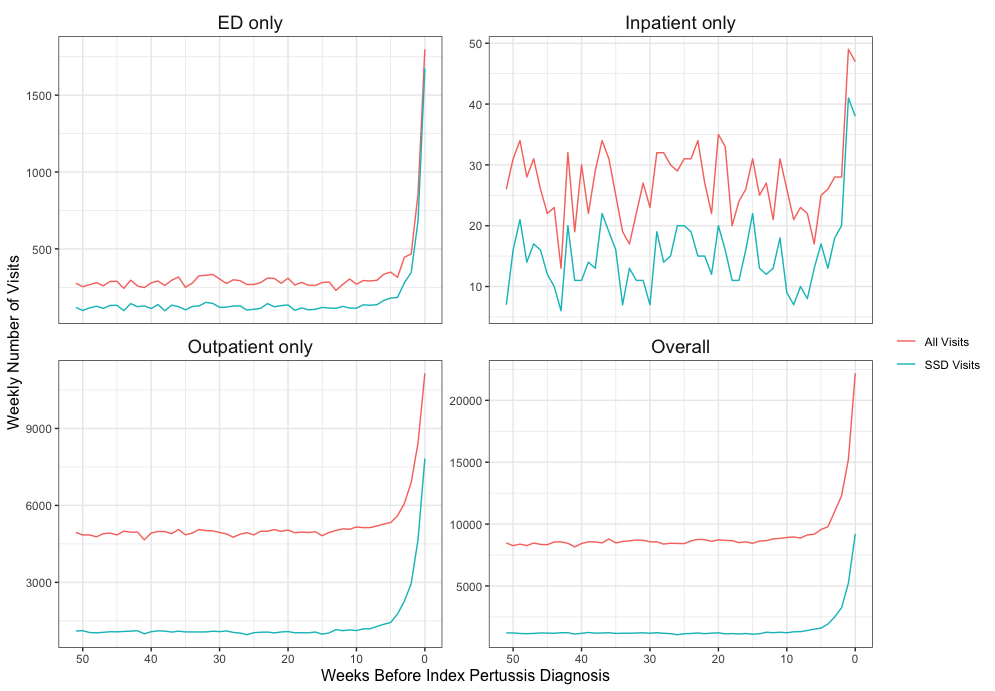

Supplement: Supplementary file 1 [file S0899823X23000314sup001.docx]
